# Beyond GLP‐1

**DOI:** 10.1111/1753-0407.70204

**Published:** 2026-03-05

**Authors:** Zachary Bloomgarden

**Affiliations:** ^1^ Department of Medicine, Division of Endocrinology, Diabetes and Bone Disease Icahn School of Medicine at Mount Sinai New York New York USA

1

The prevalence of obesity in the United States increased from 19.3% of the adult population in 1990 to 42.5% in 2022, with a forecasted increase to 46.9% in 2035 [1]; globally the trends appear similar [2]. The glucagon‐like peptide‐1 receptor agonists (GLP1‐RAs) have shown remarkable success in treating overweight and obesity, with 12% of adults in the United States said to have at least temporarily received one of these agents [3]. However, a number of issues should be considered in GLP‐1RA use. Not all treated individuals have the desired degree of weight loss; this is particularly an issue for people with type 2 diabetes, for whom the degree of weight loss is less than that among people not having diabetes [4]. Gastrointestinal intolerance prevents a sizable number of people from using these agents or necessitates their use in lower doses. We have reviewed the question as to whether weight loss‐associated decrease in muscle mass may lead to clinical sarcopenia, in turn associated with frailty and susceptibility to a variety of adverse consequences [5]. It is likely that these considerations are relevant to important subsets of treated individuals as we further extend weight control agents to address more chronic illnesses. The development of novel approaches to attain weight loss, with greater gastrointestinal tolerability, and without reduction in muscle mass should then be seen as a priority. The approaches being evaluated span a variety of different mechanisms. Their use will almost certainly be developed in conjunction with GLP‐1RAs, potentially allowing more tolerable dosing and a greater degree of weight loss in.

Over the coming issues of the Journal of Diabetes, we will review some potential adjunctive agents that may find efficacy in combination with the GLP‐1RA in an effort to stimulate discussion of these approaches (Figure 1). We look forward to contributions to the Journal addressing these approaches.

**FIGURE 1:**
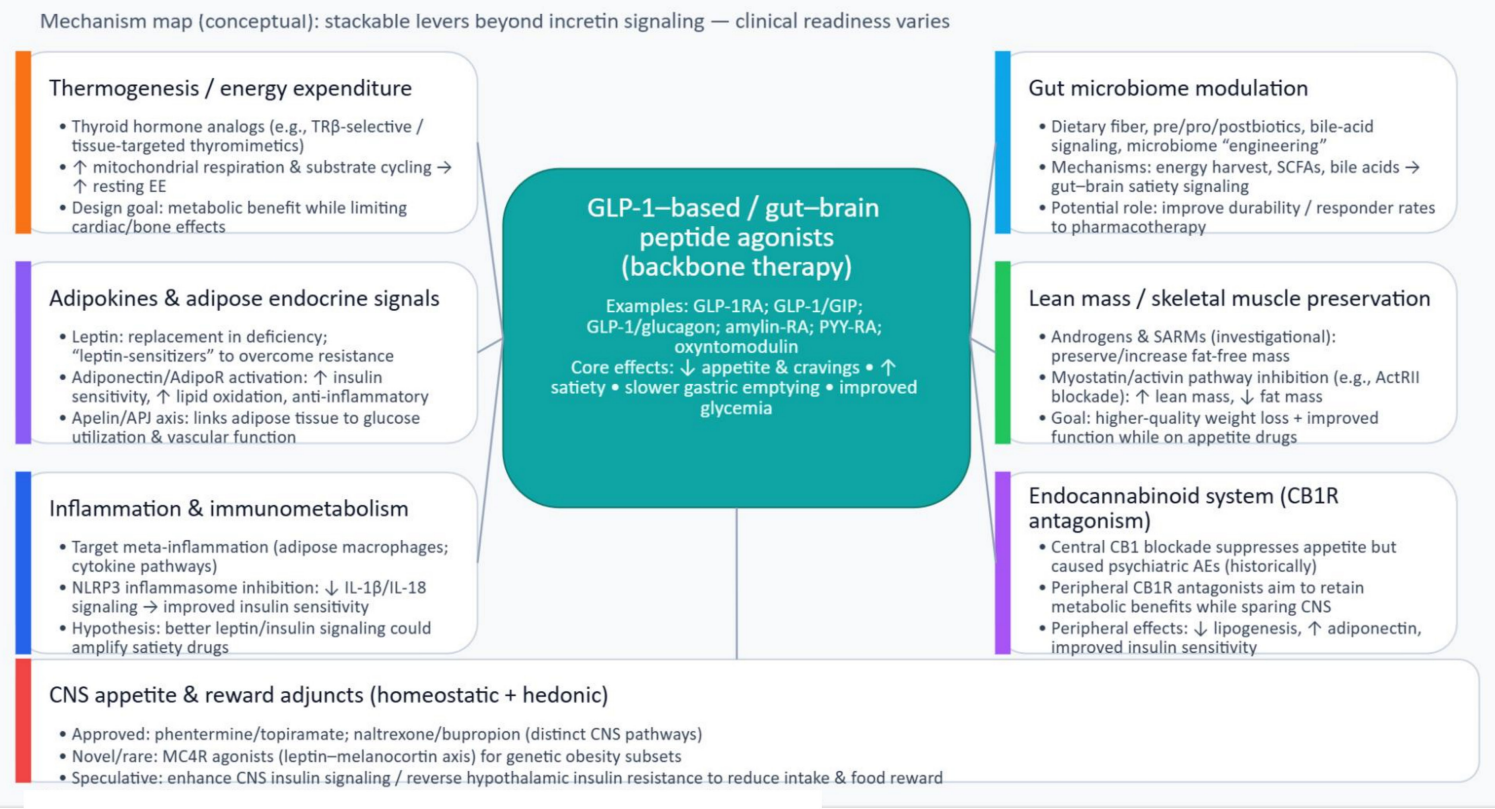
Non‐incretin adjunct targets that could complement GLP‐1 / multi‐agonists in obesity.

## Funding

The author has nothing to report.

## Conflicts of Interest

The author declares no conflicts of interest.
